# Housing difficulties, health status and life satisfaction

**DOI:** 10.3389/fpsyg.2022.1024875

**Published:** 2022-12-21

**Authors:** Mingzhi Hu, Yinxin Su, Xiaofen Yu

**Affiliations:** ^1^Department of Engineering Management, School of Management, Chinese Academy of Housing and Real Estate, Zhejiang University of Technology, Hangzhou, Zhejiang, China; ^2^Department of Urban Development and Management, College of Public Affairs, Zhejiang University, Hangzhou, Zhejiang, China

**Keywords:** housing difficulties, life satisfaction, physical health, psychological health, mechanisms

## Abstract

This study examines the effects of housing difficulties on life satisfaction. By using longitudinal data from the China Family Panel Studies survey, we find strong evidence that households who experience housing difficulties are less satisfied with their lives than those who do not after controlling for a wide range of household demographic and socioeconomic characteristics and county and year fixed effects. Our estimated results are robust to unobservable household characteristics, model misspecification and selection bias. We also provide explanations for the negative effects of housing difficulties on life satisfaction through which housing difficulties are detrimental to physical and psychological health. Life satisfaction remains negatively associated with housing difficulties even after controlling for health status.

## Introduction

Housing has been described as the foundation of social care (Windle et al., 2006) and considered a significant determinant of health and wellbeing (Hu, 2013; Zumbro, 2014; Huang et al., 2015; Ren et al., 2018; Hu and Ye, 2020). The home environment is of tremendous significance to human beings, as the residential setting is where people typically spend most of their time and contact with the most important members of one’s social network (Evans et al., 2003). Thus, housing difficulties are corollaries of poverty and threats to family wellbeing (Courtney et al., 2004). The loss of low-income housing and the growth in the urban poverty population have created a situation in which some are destined to be homeless or without adequate housing (Wright and Rubin, 1991). A release from the Office for National Statistics, which is the United Kingdom’s largest independent producer of official statistics, considered housing difficulties as any periods of time when individuals had no home of their own (either rented or owned) and summarised four types of homelessness: staying with friends or relatives temporarily, staying in emergency or other temporary accommodation, staying in a place not intended as a permanent home and ‘rough sleeping’ or sleeping in a public place (see, “Past experiences of housing difficulties in the United Kingdom: 2018” on October 22, 2020 by Mark Hamilton and Ben Hayes at https://www.ons.gov.uk/).

The essence of housing difficulties is the lack of affordable housing with suitable living environment driven by an increase in property prices and a decrease in relative wages. Empirically, housing difficulties are measured in many aspects that reflect inadequate housing and living conditions. For example, Windle et al. (2006) interviewed a random sample of older people in Wales to investigate whether they had difficulties with items, such as difficulties with steps/stairs; heating, damp/condensation and draughts; and difficulties using bath/shower and water closet. As pointed out by Windle et al. (2006), these self-reported housing difficulties can be exacerbated by reduced physical functioning, especially for older adults. From the perspective of housing unaffordability, Courtney et al. (2004) considered a household has housing difficulties if the household at any time in the previous 12 months had not enough money to pay the rent or mortgage, been evicted, moved in with family or friends or been homeless for at least one night. Similarly, Marques et al. (2014) suggested that housing difficulties mainly include derelict buildings, rundown social housing districts, overcrowded dwellings, houses lacking basic amenities and houses without the minimum living conditions. In line with previous studies, we consider the family to have housing difficulties if any of the following conditions exist: children over age 12 live in the same room with the parents, family members of three generations live in the same room, children of different genders over age 12 live in the same room, beds are laid out at night and folded up during the daytime, beds are laid out in the living room and other difficulties.

This study generates three contributions to the literature. First, it identifies an undetected factor, namely, housing difficulties, as a new antecedent of individuals’ subjective wellbeing and thus sheds light on the sources of diversity in the subjective wellbeing across households with different living and housing conditions. Sociologists and economists have substantially discussed the effects of many social and economic factors on the subjective wellbeing of citizens (Shields and Price, 2005; Diego-Rosell et al., 2018). The real estate literature also pays considerable attention to the causes of subjective wellbeing (Cattaneo et al., 2009; Nakazato et al., 2011; Zhang et al., 2018; Zhang and Zhang, 2019; Hu et al., 2020) but focuses mainly on the influence of homeownership status (Hu, 2013; Zumbro, 2014; Cheng et al., 2016; Ren et al., 2018; Hu and Ye, 2020). Homelessness is unquestionably a housing problem (Wright and Rubin, 1991) and is even considered the most serious housing problem (Courtney et al., 2004). This study extends this line of research by investigating the effects of housing difficulties—an important yet ignored factor. Drawing on data collected by the China Family Panel Studies (CFPS) survey, we find strong evidence that households who experience housing difficulties are less satisfied with their lives than those who do not after controlling for a wide range of household demographic and socioeconomic characteristics and county and year fixed effects. Our estimated results are robust to unobservable household characteristics, model misspecification and selection bias. Given that life satisfaction is an important component of subjective wellbeing (Diener and Chan, 2011; Ma et al., 2017), our results suggest that housing difficulties are a reliable antecedent of individuals’ subjective wellbeing.

Second, by extending the outcomes caused by housing difficulties to household wellbeing, this study advances our knowledge on the implications of housing improvements. Existing studies have found that housing improvements are associated with variations in health and associated socioeconomic outcomes (for an excellent review, see Thomson et al. (2013)). Following this line of research, this study extends the effects of housing difficulties on individuals’ life satisfaction. We find that housing difficulties strongly affect life satisfaction amongst households, such that households suffering from housing difficulties are more likely to report a lower score of life satisfaction. This finding not only enriches our understanding about the consequences of housing difficulties but also carries rich practical implications.

Third, this study enhances our understanding of the possible mechanisms underlying the relationship between housing difficulties and household wellbeing. There exist various mechanisms through which housing difficulties might affect individuals’ life satisfaction. This study provides a possibility that the negative effects of housing difficulties on life satisfaction work through which housing difficulties lead to worse health status. Previous studies have documented that housing difficulties predict poorer health status (Krieger and Higgins, 2002; Windle et al., 2006; Cattaneo et al., 2009; Navarro et al., 2010; Palacios et al., 2020). In line with the relevant research, we find that housing difficulties pose a threat to physical and psychological health. Moreover, life satisfaction is negatively associated with poor health status. We also find that the negative relationship between housing difficulties and life satisfaction holds after we control for physical and psychological health, thereby suggesting that housing difficulties may affect life satisfaction through other channels. Hence, this study identifies a possible channel by which housing difficulties might matter and calls for future studies offering deep insight into other channels.

The remaining part of this paper is organised as follows. In the next section, we develop the research hypothesis that housing difficulties have negative effects on life satisfaction after a brief literature review on related studies. In Section 3, we discuss the data and present summary statistics. In Section 4, we provide the baseline empirical results of the effects of housing difficulties on life satisfaction. In Section 5, we provide two possible explanations that housing difficulties affect life satisfaction. In Section 6, we conduct two robustness checks related to omitted variable bias, model misspecification and sample selection bias. In the last two sections, we provide a brief discussion of our results and draw conclusions with remarks.

## Literature review

### General determinants of life satisfaction

Individuals’ subjective wellbeing is increasingly considered more important than other economic indicators, such as income and wealth to measure individual and societal welfare (Dolan and White, 2007). The question of what determines individuals’ subjective wellbeing has been extensively researched in the literature. Subjective wellbeing is often defined as being satisfied with one’s life whilst feeling good, and this conceptualisation also involves cognitive and affective appraisals of life (Veenhoven, 2012). Life satisfaction is an overall assessment of feelings and attitudes about one’s life at a particular point in time ranging from negative to positive (Prason and Chaturvedi, 2016), and happiness is commonly considered a profound mental state of satisfaction and contentment (Lu, 2001). Life satisfaction and happiness are often used to assess subjective wellbeing in empirical studies (Hu, 2013; Zhang et al., 2018; Hu et al., 2020; Hu and Ye, 2020). The literature on wellbeing uses terms such as happiness, life satisfaction and subjective wellbeing interchangeably (Diener et al., 2002).

The general determinants of life satisfaction include household demographic characteristics (e.g., age, gender and marital status) and socioeconomic characteristics (e.g., income and education), occupation and social status, opportunities and social mobility, welfare provision, government policy, social networks and family tradition, neighbourhood environment and housing tenure (Appleton and Song, 2008; Hu, 2013; Zumbro, 2014; Ma et al., 2017; Ren et al., 2018; Hu et al., 2020; Hu and Ye, 2020). Ma et al. (2017) developed a comprehensively analytical framework to examine the determinants of life satisfaction, including objective and subjective measures. A recent study by Hu et al. (2020) summarises the determinants of individuals’ happiness into three categories: demographic characteristics, socioeconomic factors and social factors. Diener et al. (2002) and Prason and Chaturvedi (2016) provided excellent reviews on the factors associated with life satisfaction and some related issues. We add to this line of research by studying the effects of housing difficulties on life satisfaction, which is considered one of the most important measurements of wellbeing.

### The role of housing difficulties

Plenty of evidence suggests that housing is an important determinant of social and economic outcomes (Aaronson, 2000; Dietz and Haurin, 2003; Engelhardt et al., 2010; Coulson and Li, 2013). Homeownership brings many economic benefits, such as wealth accumulation (Turner and Luea, 2009), mortgage interest deduction (Bourassa et al., 2013) and increased consumption (Chen et al., 2020), as well as social benefits, such as better citizenship (DiPasquale and Glaeser, 1999), improved self-esteem and subjective wellbeing (Hu and Ye, 2020), improved neighbourhood conditions (O’Sullivan and Gibb, 2012), higher quality of home environment, better child outcomes (Haurin et al., 2002) and other external benefits (Coulson and Li, 2013). Housing assistance policies that increase access to homeownership for low-income households are also proved to have positive effects on households’ socioeconomic outcomes and wellbeing (Beer et al., 2011; Van Dijk, 2019).

However, far too little attention has been paid to the role of housing difficulties in affecting individuals’ wellbeing. Several studies tangentially touch on the issues. For example, Cattaneo et al. (2009) examined the effect of a large-scale Mexican program that replaces dirt floors with cement floors on child health and adult happiness, and results showed that this program results in a significant improvement in children’s cognitive development and adult welfare measured by increased satisfaction with their housing and quality of life. Zhang et al. (2018) studied the association between the average housing and overall satisfaction levels and several housing characteristics, such as house size, number of bedrooms, the existence of living rooms or bathrooms and housing type. The empirical results by Zhang et al. (2018) showed that all house-related characteristics significantly affect individuals’ housing satisfaction, whereas only homeownership and house size matter for overall happiness. Hu et al. (2022) documented that housing quality, measured by housing value and physical housing conditions, plays an important role in determining individuals’ overall happiness by using data from a large-scale survey in China. Oswald et al. (2003) examined the role of housing-related variables in affect life satisfaction of older adults in Germany. This study differs from the aforementioned works in two aspects. Our study differs from the aforementioned works in two aspects. First, we focus on housing difficulties rather than physical housing conditions. Housing conditions are not the same as housing difficulties. Whether a family has housing difficulties is usually defined in terms of the family’s housing conditions. Relatively poor housing conditions do not necessarily imply housing difficulties. Relatively poor housing conditions do not necessarily imply housing difficulties. Similarly, relatively good housing conditions do not necessarily mean the absence of housing difficulties. For example, households with large-sized houses can be considered as having good housing conditions; however, they could be also considered as having housing difficulties if they have a large family. It is therefore not surprising that Zhang et al. (2018) showed an insignificant impact of most house-related characteristics (i.e., housing conditions) on happiness in China where relatively good conditions already exist. Second, we identify two mechanisms through which housing difficulties are detrimental to physical and psychological health. Nevertheless, we do not deny that housing difficulties affect life satisfaction through other channels.

Researchers have shown an increased interest in the connection between housing difficulties and health (Smith and Mallinson, 1997; Allen, 2000; Krieger and Higgins, 2002; Cattaneo et al., 2009; Navarro et al., 2010; Cleland et al., 2016; Xie, 2019; Palacios et al., 2020). Housing deprivation, such as lack of certain facilities, structural problems and overcrowding, not only reflects a failure of a basic functioning but also poses a serious physical and mental health threat (Navarro et al., 2010). Housing-related events, such as moving home and home improvements, are important for residents’ health, although they do not happen as frequently as other life events (Cleland et al., 2016). There exists almost sure a strong consensus that poor housing is associated with ill health. For example, by using a population sample of older people in Wales, Windle et al. (2006) found that housing-related difficulties, specifically being cold with current heating and hours spent at home, lead to poorer health status. Palacios et al. (2020) also found that individuals who live in homes with poor housing conditions on average have worse mental and physical health and experience increased doctor visits by using a dataset from a long-running German panel survey. Poor housing conditions may cause a wide range of health problems, including respiratory infections, asthma, lead poisoning, injuries and mental health (Krieger and Higgins, 2002). Overall, these findings indicate a possible channel for the negative effects of housing difficulties on individuals’ wellbeing; that is, housing difficulties lead to poor health.

Obtaining the estimated effects of housing difficulties in China is of academic and policy importance for several reasons. First, China has transformed rapidly from a country with a welfare-oriented housing system to a country with one of the highest homeownership rates. The housing market in China has experienced a persistent and unprecedented boom since the privatisation reform in 1998 that allowed public housing tenants to buy their state-owned housing units at heavily subsidised prices (Chen et al., 2019, 2022). Homeownership rate has surged from less than 30% to more than 80% within one decade after the reform (Chen and Hu, 2019). The average homeownership rate in China reached almost 90% in 2014, which is considerably higher than that in the Euro area (66.4%) or the United States (64.2%; Wang and Zhang, 2020). Second, housing prices and housing wealth have been growing rapidly in China during the last two decades. Subsequent to the reform, China’s housing prices have been growing nearly twice as fast as the national income over the past decade (Chen and Wen, 2017). According to the data of the National Bureau of Statistics of China, the average housing price grew 18% annually from 2000 to 2020. Housing assets currently constitute by far the dominant portion of total household wealth (Chen et al., 2020). Third, housing conditions are becoming increasingly differentiated in China, although the overwhelming majority of households in this country own their homes. Significant disparities also exist in housing conditions and neighbourhood environments amongst Chinese homeowners in different neighbourhoods, regions and generations (Wang and Zhang, 2020). Although the degree of housing differentiation between different socioeconomic groups is high, the differentiation within each group is even more significant (Liu et al., 2012).

## Data

We construct a panel dataset by using data from the CFPS, a nationally representative and longitudinal household survey that started in 2010 with a sample of almost 30,000 individuals living in almost 15,000 families, for an approximate response rate of 79%.^2^ Individuals in each household were followed biennially from 2010 to 2018. We use the first three waves of (i.e., 2010, 2012 and 2014) the survey data because the information on housing difficulties is not available after the 2014 survey. We define the independent variable as an indicator variable of housing difficulties, which equals 1 if there exists at least one type of housing difficulties and 0 otherwise.

**Figure 1:**
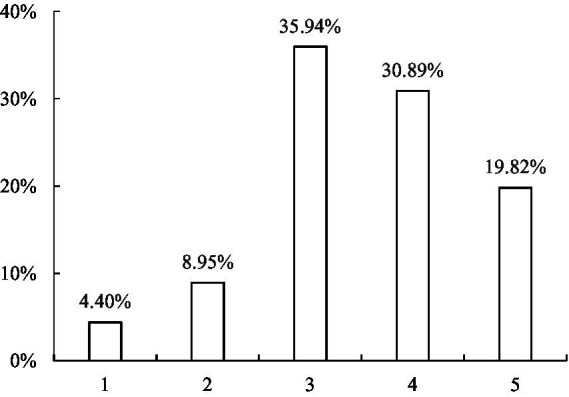
Frequency distribution of life satisfaction for the sample. Life satisfaction is measured on a 5-point scale, ranging from 1 (very unsatisfied) to 5 (very unsatisfied). Data source: China Family Panel Studies 2010, 2012, and 2014.

The CFPS also interviews about the life satisfaction of each respondent. Specifically, the respondents are asked how satisfied they are with their lives, and the choices for this question are arranged on a five-point Likert scale ranging from 1 to 5, in which 1 represents ‘very unsatisfied’ and 5 represents ‘very satisfied’. On the basis of this survey question, we define the dependent variable as an ordered variable of satisfaction with life. The frequency distribution of life satisfaction is shown in Figure 1, which shows that life satisfaction has a nearly bell-shaped frequency distribution that is slightly skewed to the right. Overall, the distribution of life satisfaction is consistent with that reported in previous studies (Appleton and Song, 2008; Zumbro, 2014; Ren et al., 2018; Collischon, 2019).

The dataset also contains detailed household demographic information (i.e., gender, age, marital status and geographic location) and socioeconomic characteristics (i.e., education, medical insurance, income, deposit, wealth and debt). These details allow us to control for not only the common household characteristics affecting the overall life satisfaction (Chen et al., 2015; Ren et al., 2018; Zhang et al., 2018) but also several unique political and social variables with Chinese characteristics, such as political and hukou status.^3^

The initial dataset contains 155,980 observations (57,155 in 2010, 61,423 in 2012 and 65,499 in 2014). Several screenings are applied to the sample in our analysis. First, we restrict our attention to the urban sample because housing is tradable only amongst villagers, and the housing market is thus nearly nonexistent in rural China, although the survey contains urban and rural households. Second, we discard observations with missing value for the variables used in the econometric analysis. Third, observations with abnormal values (e.g., self-reported score of life satisfaction less than 1 and more than 5; and household income, deposit, financial asset value, housing asset value and debts less than 0) are dropped. The final dataset is an unbalanced panel with 39,987 observations, with 14,853 observations in the baseline survey and 13,413 and 11,721 observations in the following two waves of surveys.

Table 1 provides the summary statistics for the groups of households with housing difficulties and households without housing difficulties, as well as for the full sample. The variables shown in Table 1 include life satisfaction and household characteristics from the CFPS. All these household characteristics are well controlled in our econometric regressions. In the dataset, 15.9% of the households are reported to have experienced housing difficulties. The average score of life satisfaction is 3.582, but it varies significantly between households with and without housing difficulties. Specifically, we find that the average score of life satisfaction for households with housing difficulties is 3.312, which is 0.256 less than that for households without housing difficulties. This preliminary finding suggests that housing difficulties may decrease life satisfaction. However, the two groups have clear differences in many aspects of household demographic and socioeconomic characteristics. For example, households with more income, deposit and wealth tend to be less likely to have housing difficulties. These systematic differences suggest that econometric analysis that controls for these household characteristics is necessary.

**Table 1:** Summary statistics.

Variables	Full sample	Households with housing difficulties	Households without housing difficulties	The mean differences	*p*-value
Housing difficulties	0.159				
Life satisfaction	3.528	3.312	3.568	−0.256	0.000
**Demographic characteristics**					
Female	0.521	0.526	0.520	0.006	0.126
Age	46.14	45.16	46.32	−1.163	0.001
Urban hukou	0.543	0.588	0.535	0.053	0.000
Communist	0.298	0.295	0.299	−0.004	0.003
Married	0.821	0.792	0.827	−0.035	0.000
**Socioeconomic status**					
*Education*					
Primary school and below	0.254	0.256	0.254	0.002	0.079
Middle & high school	0.511	0.521	0.510	0.011	0.645
Three-year college	0.145	0.148	0.145	0.003	0.021
Four-year college and above	0.089	0.075	0.092	−0.017	0.928
Insurance	0.805	0.753	0.815	−0.062	0.000
Household income	4.961	4.358	5.075	−0.717	0.017
Household deposit	3.133	2.236	3.302	−1.066	0.000
Finance assets value	0.898	0.888	0.900	−0.012	0.672
Housing assets value	40.88	25.38	43.81	−18.43	0.000
Household debts	0.872	0.949	0.858	0.091	0.002
Observations	39,987	6,352	33,635		

Data source: China Family Panel Studies (CFPS) 2010, 2012 and 2014. Housing difficulties is an indicator variable of housing difficulties, which includes children over age 12 live in the same room with the parents, family members of three generations live in the same room, children of different genders over age 12 live in the same room, beds are laid out at night and folded up during the daytime, beds are laid out in the living room and other difficulties; Life satisfaction is an ordered variable of satisfaction with life, which is measured on a 5-point scale ranging from 1 to 5 (“1” is lowest, and “5”is highest); Female is an indicator variable of female individuals; Age is a continuous variable of the age of individuals; Urban hukou is an indicator variable of individuals with a urban hukou; Communist is an indicator variable of individuals being the member of the Communist Party of China; Married is an indicator variables of individuals being married; Education refers to the highest educational attainment. Insurance is an indicator variable of individuals having medical insurance. Household income, measured as 10,000 yuan, refers to the total household income for the past 12 months; Household deposit, measured as 10,000 yuan, refers to the total amount of deposits currently held by the family; Finance assets value, measured as 10,000 yuan, refers to the total value of financial products owned by the family; Housing assets value, measured as 10,000 yuan, refers to the total value of housing assets owned by the family; Household debts, measured as 10,000 yuan, refers to the total amount of money that the family owes to others.

## Main empirical results

We use the ordered logit model to examine the effects of housing difficulties on individuals’ life satisfaction because of the ordinal outcome of life satisfaction in this study. The results are robust to various estimation methods, as we will discuss in the section of robustness checks. We initiate the following form of baseline ordered logit model:


(1) $$\begin{array}{c} \mathrm{Life}\;\mathrm{satisfactio}n_{ijt}=\beta_{0,1}+\beta_{1,1}\mathrm{Housing}\;\mathrm{difficultie}s_{ijt}\\ \;+X_{ijt}+\theta_{j}+\delta_{t}+\varepsilon_{ijt}\text{,} \end{array}$$

where the dependent variable $\mathrm{Life}\;\mathrm{satisfactio}n_{ijt}$ is an ordered variable that denotes individual *i*’s overall satisfaction with life in county *j* and year *t*. Overall life satisfaction is measured on a five-point scale ranging from 0 (very unsatisfied) to 5 (very satisfied). The independent variable $\mathrm{Housing}\;\mathrm{difficultie}s_{ijt}$ is an indicator variable, which equals 1 if individual *i* has housing difficulties in county *j* and year *t* and 0 otherwise. We are particularly interested in the coefficient of $\mathrm{Housing}\;\mathrm{difficultie}s_{ijt}$ , which is expected to be negative and statistically different from zero. $X_{ijt}$ is a vector of control variables, including household characteristics summarised in Table 1. County dummies ( $\theta_{j}$ ) and year dummies ( $\delta_{t}$ ) are also added in the model to immunise our estimations from the effects of time-invariant regional characteristics at the county level and time series trend. Finally, $\varepsilon_{ijt}$ is the error term.

We estimate four specifications by gradually increasing the number of controlled variables to see their effects on life satisfaction and check the robustness of the coefficient of housing difficulties. The estimated coefficients, standard errors and significance levels are reported in Table 2. Column (1) of Table 2 reports the results with the simplest specification by controlling for housing difficulties only. As expected, the results indicates that without controlling for any observables, households facing housing difficulties are less satisfied with their lives than those who do not. This effect is around 0.433 point in the transformed life satisfaction scale and statistically significant at the 1% level.

**Table 2:** Impact of housing difficulties on life satisfaction (ordered logit regressions).

	(1)	(2)	(3)	(4)
Housing difficulties	−0.433***	−0.424***	−0.351***	−0.300***
	(0.027)	(0.027)	(0.027)	(0.028)
**Demographic characteristics**				
Female		0.147***	0.155***	0.130***
		(0.018)	(0.018)	(0.019)
Age		−0.076***	−0.080***	−0.080***
		(0.004)	(0.004)	(0.004)
Age-squared		0.001***	0.001***	0.001***
		(0.000)	(0.000)	(0.000)
Urbanhukou		−0.018	−0.112***	−0.038
		(0.018)	(0.021)	(0.026)
Communist		0.715***	0.645***	0.198***
		(0.020)	(0.023)	(0.036)
Married		0.341***	0.319***	0.270***
		(0.031)	(0.031)	(0.032)
**Socioeconomic status**				
Education (Ref.: Primary school and below)				
Middle & high school			0.045*	−0.019
			(0.026)	(0.027)
Three-year college			0.026	−0.056
			(0.036)	(0.039)
Four-year college and above			0.017	−0.034
			(0.040)	(0.043)
Insurance			0.166***	0.145***
			(0.025)	(0.026)
Ln(Household income)			0.144***	0.210***
			(0.013)	(0.014)
Ln(Household deposit)			0.068***	0.109***
			(0.010)	(0.011)
Ln(Finance assets value)			0.003**	0.002**
			(0.001)	(0.001)
Ln(Housing assets value)			0.041***	0.060***
			(0.006)	(0.007)
Ln(Household Debts)			−0.077***	−0.074***
			(0.015)	(0.016)
**Other variables controlled**				
Year dummies	No	No	No	Yes
County dummies	No	No	No	Yes
Pseudo *R*^2^	0.0026	0.0215	0.0262	0.0446
Observations	39,987	39,987	39,987	39,987

*Significant at 10% level, **significant at 5% level, ***significant at 1% level.

In Column (2) of Table 2, we further control for household demographic variables, including gender, age and its squared term, hukou status, political status and marital status. The results show that the coefficient of housing difficulties only changes slightly from −0.433 to −0.424 and remains significant at the 1% level. Consistent with findings in the previous literature, we find that females and married couples are more satisfied with their lives (Cheng et al., 2016; Zhang and Zhang, 2019; Hu and Ye, 2020). A U-shaped relationship is also noted between age and life satisfaction (Hu, 2013; Zhang et al., 2018; Zhang and Zhang, 2019). As expected, communists have higher levels of life satisfaction than others (Cheng et al., 2016; Hu et al., 2020; Hu and Ye, 2020).

Column (3) of Table 2 provides the results by further controlling for household socioeconomic characteristics, including education, insurance, household income, household deposit, finance asset value, housing asset value and household debts. When these controls are added, the coefficient of housing difficulties on life satisfaction becomes −0.351 and remains significant at the 1% level. The effects of household socioeconomic characteristics are as in line with previous studies. For example, people with higher income and wealth are more satisfied with their lives (Borooah, 2006; Ren et al., 2018; D’Ambrosio et al., 2019).

The last column of Table 2 shows the results by further controlling for year and county fixed effects. The coefficient of housing difficulties slightly changes to −0.300 and remains statistically significant at the 1% level. Overall, the results reported in Columns (1)–(4) of Table 2 suggest persistent and negative impacts of housing difficulties on life satisfaction.^4^

## Possible explanations

We observe in our data that housing difficulties decrease life satisfaction. We now provide and test two potential explanations on this finding. One is that living in a home with poor quality poses a threat to one’s physical health, which can negatively affect life satisfaction. The second possibility is that housing difficulties is detrimental to one’s psychological health. Overall, we find that housing difficulties harm physical and psychological health, which decreases life satisfaction, whereas the negative relationship between housing difficulties and life satisfaction still holds after we control for household physical health.^5^

### Housing difficulties, physical health and life satisfaction

The real estate literature has documented that poor living quality is detrimental to physical health. Our data also show that households living in a poor-quality home are more likely to suffer from physical health problems. Specifically, 17.5% of households with housing difficulties are reported to have a poor health, whereas only 13.3% of households without housing difficulties rate their physical health as poor. Unconditionally, households with housing difficulties are 2.2 and 3.6 percentage points more likely to have recently experienced chronic disease and bad memory, respectively, than households without housing difficulties. All these differences are statistically significant at the 1% level.

We commence empirical investigations on whether housing difficulties pose a threat to physical health and whether poor physical health is negatively associated with life satisfaction when all other things are equal by using the following two regression models:


(2) $$\begin{array}{c} \mathrm{Physical}\;\mathrm{healt}h_{ijt}=\beta_{0,2}+\beta_{1,2}\mathrm{Housing}\;\mathrm{difficultie}s_{ijt}\\ \;+X_{ijt}+\theta_{j}+\delta_{t}+\varepsilon_{ijt}\text{,} \end{array}$$

(3) $$\begin{array}{c} \mathrm{Life}\;\mathrm{satisfactio}n_{ijt}=\beta_{0,3}+\beta_{1,3}\mathrm{Housing}\;\mathrm{difficultie}s_{ijt}\\ \;+\beta_{2,3}\mathrm{Physical}\;health_{ijt}+X_{ijt}+\theta_{j}\\ \;+\delta_{t}+\varepsilon_{ijt}\text{,} \end{array}$$

where $\mathrm{Physical}\;\mathrm{healt}h_{ijt}$ represents individual *i*’s physical health in county *j* and year *t* and takes several outcomes, that is, the three indicator variables of poor health condition, chronic disease and bad memory. Other variables are defined the same as those in Eq. (1). We use the logit model to estimate Eq. (1) due to the binary outcome of physical health and the ordered logit model to estimate Eq. (2).

Columns (1)–(3) of Table 3 present the results from Eq. (2) by running separate regressions with the same set of control variables but with different dependent variables: an indicator variable of poor health condition, an indicator variable of chronic disease and an indicator variable bad memory in sequence. The results show that throughout Columns (1)–(3), the coefficient of housing difficulties remains positive and statistically significant at the 1% level, indicating that all other things being equal, households with housing difficulties are more likely to have a poor health condition, chronic disease and bad memory. Column (4) of Table 3 shows the results estimated from Eq. (3) by adding the three indicator variables of physical health. Two points are worth noting here. First, the results show that the coefficient of poor health condition, chronic disease and bad memory is negative and statistically significant, suggesting a negative effect of poor physical health on life satisfaction. Second, we consistently find a negative and statistically significant coefficient of housing difficulties after controlling for physical health. Overall, the results in Table 3 provide supportive evidence for the first explanation that poor physical health resulting from living in poor quality or unsuitable housing decreases life satisfaction.

**Table 3:** Housing difficulties, physical health and life satisfaction.

	(1)	(2)	(3)	(4)
	Poor health condition	Chronic disease	Bad memory	Life Satisfaction
Housing difficulties	0.310***	0.173***	0.217***	−0.278***
	(0.042)	(0.042)	(0.039)	(0.028)
**Physical health**				
Poor health condition				−0.584***
				(0.033)
Chronic disease				−0.151***
				(0.029)
Bad memory				−0.152***
				(0.028)
**Other variables controlled**				
Demographic characteristics	Yes	Yes	Yes	Yes
Socioeconomic status	Yes	Yes	Yes	Yes
Year dummies	Yes	Yes	Yes	Yes
County dummies	Yes	Yes	Yes	Yes
Pseudo *R*^2^	0.1423	0.1115	0.1309	0.0496
Observations	39,987	39,987	39,987	39,987

*Significant at 10% level, **significant at 5% level, ***significant at 1% level. Households demographic characteristics include gender, age, hukou status, political status and marital status. Household socioeconomic status includes education, insurance, household income, household deposit, finance assets value, housing assets value and household debts.

### Housing difficulties, psychological health and life satisfaction

We examine the second explanation that living in a home with poor quality is not conducive to psychological health and thus reduces life satisfaction. We use three variables to proxy for psychological health: an indicator variable of respondents who feel depressed that nothing can cheer them up in the past month (*Depress*), an indicator variable of respondents who feel that everything is an effort in the past month (*Difficult*) and an indicator variable of respondents who feel hopeless in the past month (*Hopeless*). Our data show that unconditionally, households with housing difficulties are 3.4, 3.2 and 2.4 percentage points more likely to feel depressed, feel that everything is an effort and feel hopeless frequently in the past month, respectively, than households without housing difficulties. These findings provide preliminary evidence that housing difficulties are harmful for psychological health.

We now examine the role of psychological health in the relationship between housing difficulties and life satisfaction by using the following two regressions:


(4) $$\begin{array}{c} \mathrm{Psychological}\;\mathrm{healt}h_{ijt}=\beta_{0,4}+\beta_{1,4}\mathrm{Housing}\;\mathrm{difficultie}s_{ijt}\\ \;+X_{ijt}+\theta_{j}+\delta_{t}+\varepsilon_{ijt}\text{,} \end{array}$$

(5) $$\begin{array}{c} \mathrm{Life}\;\mathrm{satisfactio}n_{ijt}=\beta_{0,5}+\beta_{1,5}\mathrm{Housing}\;\mathrm{difficultie}s_{ijt}\\ \;+\beta_{2,5}\mathrm{Psychological}\;\mathrm{healt}h_{ijt}\\ +X_{ijt}+\theta_{j}\;+\delta_{t}+\varepsilon_{ijt}\text{,} \end{array}$$

where $\mathrm{Psychological}\;\mathrm{healt}h_{ijt}$ represents individual *i*’s psychological physical health in county *j* and year *t* and takes several outcomes, which are three indicator variables of often felling depressed (*Depress*), difficult to do anything (*Difficult*) and hopeless (*Hopeless*) in the past month. Similarly, Eq. (4) is estimated using the logit model and Eq. (5) is estimated using the ordered logit model.

The results from Eq. (4) are reported in columns (1) to (3) of Table 4. The dependent variable in columns (1), (2) and (3) is *Depress*, *Difficult* and *Hopeless*, respectively. The results indicate that all other things being equal, housing difficulties increase the probability of having poor psychological health. Specifically, households with housing difficulties are more likely to feel depressed, difficult to do anything and hopeless frequently in the past month. Column (4) reports the results from Eq. (5), and the dependent variable is an ordered variable of life satisfaction. The coefficients of housing difficulties and three indicator variables of psychological health remain negative and statistically significant at the 1% level. This finding suggests that housing difficulties have a consistently negative impact on life satisfaction after controlling for psychological health and that poor psychological health decreases life satisfaction. Overall, the results in Table 4 indicate that worse psychosocial health is a possible reason that housing difficulties negatively affect life satisfaction.

**Table 4:** Housing difficulties, psychological status and life satisfaction.

	(1)	(2)	(3)	(4)
	Depressed	Difficult	Hopeless	Life Satisfaction
Housing difficulties	0.355***	0.281***	0.173**	−0.304***
	(0.054)	(0.065)	(0.075)	(0.030)
**Psychological status**				
Depressed				−0.820***
				(0.050)
Difficult				−0.297***
				(0.060)
Hopeless				−0.381***
				(0.068)
**Other variables controlled**				
Demographic characteristics	Yes	Yes	Yes	Yes
Socioeconomic status	Yes	Yes	Yes	Yes
Year dummies	Yes	Yes	Yes	Yes
County dummies	Yes	Yes	Yes	Yes
Pseudo *R*^2^	0.0574	0.0488	0.0691	0.0525
Observations	39,987	39,987	39,987	39,987

*Significant at 10% level, **significant at 5% level, ***significant at 1% level. Households demographic characteristics include gender, age, hukou status, political status and marital status. Household socioeconomic status includes education, insurance, household income, household deposit, finance assets value, housing assets value and household debts.

## Robustness checks

In this section, we address three potential issues with the estimations we have thus far: (1) Unobservable household characteristics, (2) model misspecification and selection bias and (3) measurement error.

### Unobservable household characteristics

Although we control a rich list of household variables and county and year fixed effects in previous estimations, we cannot control all factors that affect life satisfaction. Some household characteristics omitted in our previous regression analysis may explain considerably the estimated effects of housing difficulties. We attempt to ease this concern from omitted variable bias by controlling unobservable household characteristics by using panel regression models. After adding the unobservable household characteristics in the model, we can rewrite Eq. (1) as follows:


(6) $$\begin{array}{c} \mathrm{Life}\;\mathrm{satisfactio}n_{ijt}=\beta_{0,6}+\beta_{1,6}\mathrm{Housing}\;\mathrm{difficultie}s_{ijt}\\ \;+X_{ijt}+\theta_{j}+\delta_{t}+U_{i}+\varepsilon_{ijt}\; \end{array}$$

where $U_{i}$ refers to unobservable household characteristics. We use the random effect and the fixed effect models to re-estimate the coefficients. The random effect model is more efficient when time-invariant unobservables ( $U_{i}$ ) are uncorrelated with the covariates ( $X_{ijt}$ ), whereas the fixed effect model is more efficient when the two are correlated.

Columns (1) and (2) of Table 5 report the results from Eq. (6) by using random and fixed effect models. Consistent with our baseline regression, the coefficients of housing difficulties remain negative and significant in both regressions. The other covariates in the random and fixed effect estimations are similar to those in Column (4) of Table 2. That is, we control for household demographic and socioeconomic characteristics, physical health, psychological health and year and county fixed effects. In sum, our results hold after accounting for the unobserved heterogeneity of households.

**Table 5:** Impact of housing difficulties on life satisfaction (robustness checks).

	(1)	(2)	(3)	(4)
	Random-effects	Fixed-effects	PSM method	Alternative definition
Housing difficulties	−0.044**	−0.128***	−0.172***	−0.213***
	(0.022)	(0.015)	(0.018)	(0.021)
**Other variables controlled**				
Physical health	Yes	Yes	Yes	Yes
Psychological health	Yes	Yes	Yes	Yes
Demographic characteristics	Yes	Yes	Yes	Yes
Socioeconomic status	Yes	Yes	Yes	Yes
Pseudo *R*^2^	0.0962	0.0905	NA	0.0454
Observations	39,987	39,987	39,987	39,987

*Significant at 10% level, **significant at 5% level, ***significant at 1% level. Households demographic characteristics include gender, age, hukou status, political status and marital status. Household socioeconomic status includes education, insurance, household income, household deposit, finance assets value, housing assets value and household debts.

### Model misspecification and selection bias

Our main estimation on the effects of housing difficulties on life satisfaction assumes a specific model function (i.e., ordered logit model). If this model assumption is invalid, our previous estimations may be biased due to functional misspecification. To address this potential issue, we apply the propensity score matching (PSM) approach, which is a nonparametric method that does not require the choice of a functional form (Huber et al., 2015). Another issue is that households with housing difficulties tend to have lower income and wealth (as shown in Table 1), which leads to the concern of selection bias—those with housing difficulties are not comparable to those without. PSM method is also applied to address this selection bias issue.

Specifically, PSM is the identification of which compares treatment and control units with similar values on the propensity score (i.e., the conditional probability of being treated given a set of covariates), and it is a useful tool to correct for bias due to confounding variables and treatment selection. Three steps are taken to apply the PSM approach in this study. First, we regress the dependent variable that indicates whether the respondent has housing difficulties on household characteristics with a logit model. The household characteristics include gender, age, hukou status, political status, marital status, education, insurance, household income, household deposit, finance asset value, housing asset value and household debts. A propensity score for each respondent is then obtained based on the regression results. Second, we match each unit in the treatment group (households with housing difficulties) to one or more units in the control group (households without housing difficulties) by using the nearest neighbour matching with the closest propensity score. Third, we re-estimate the baseline regression, that is, Eq. (1), by using the matched samples.

The estimation results of the PSM approach are reported in Column (3) of Table 5. Again, we find that households who experience housing difficulties are less satisfied with their lives than those who do not, and the difference is statistically significant at the 1% level. This finding is consistent with our previous results. In other words, our previous results are unlikely to be driven by functional misspecification and selection bias.

The reliability of the results from PSM depends on matching quality, which can be checked whether the unconfoundedness condition and the common support condition are met. First, the unconfoundedness condition requires that the matching procedure needs to balance the distribution of the relevant variables across treatment and control groups (Rosenbaum and Rubin, 1983). We check the unconfoundedness condition by comparing the difference in the control variables for the treatment and control groups before and after matching. Our results show that the mean differences of almost all control variables decrease substantially after matching. For example, before matching, the difference in age between the treatment and control groups is 1.272; after matching, the difference substantially decreases to 0.142. This finding suggests that after matching, the control variables are balanced and comparable with respect to relevant covariates across treatment and control groups, indicating a good matching quality.

**Figure 2:**
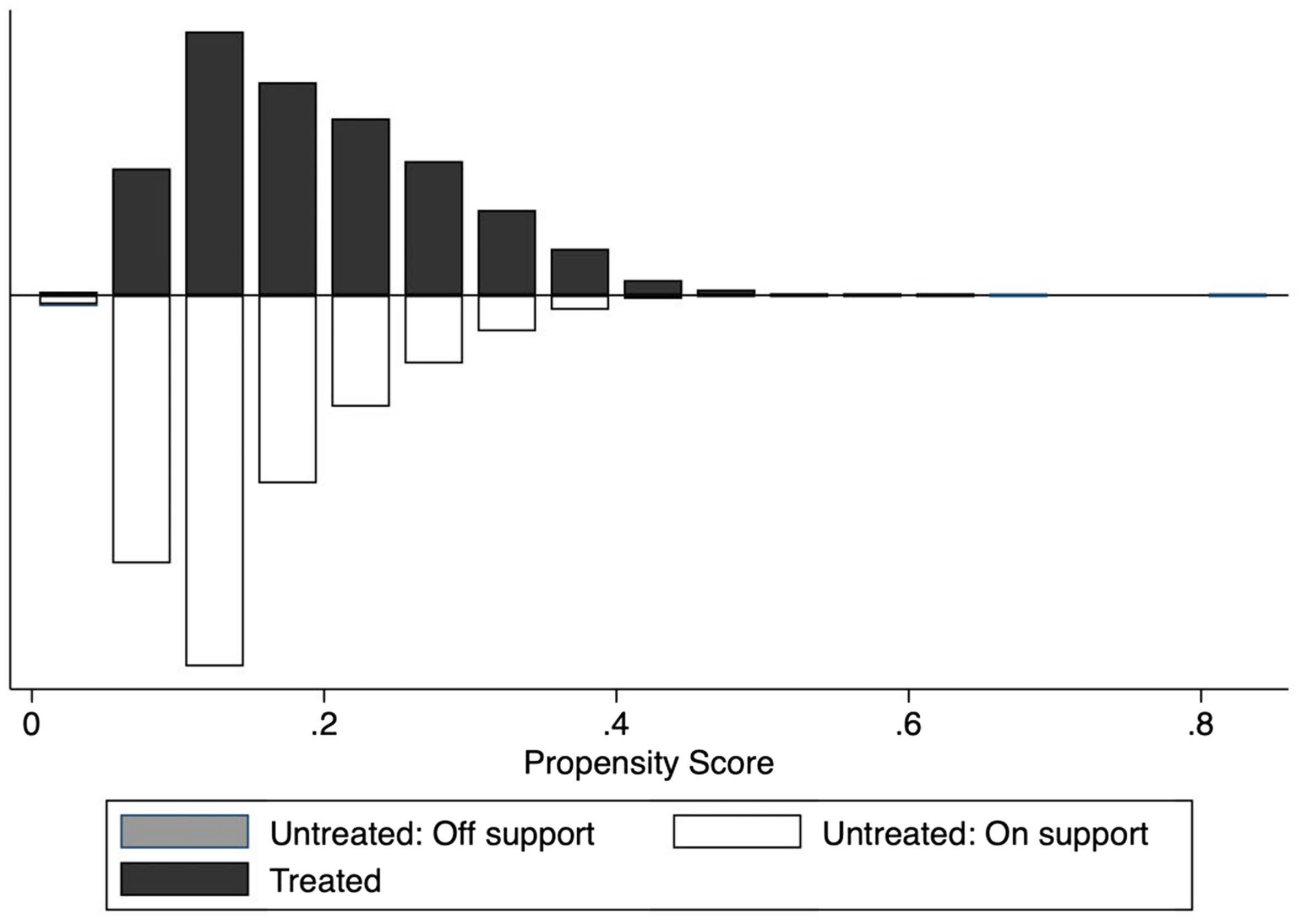
The region of common support between treated and untreated groups.

Second, PSM is a useful tool for reducing bias caused by observed confounding variables only when there exist sufficient overlaps in the distribution of propensity scores between treated and untreated groups. Figure 2 exhibits the histogram of the kernel density distribution of the propensity scores and suggests that almost all treated observations are in common support. In summary, the tests of matching quality show that our PSM estimation is reliable.

### Measurement error

In previous analyses, we define the independent variable as an indicator variable of housing difficulties. The binary measurement of housing difficulties seems arbitrary. To ease concerns over the measurement error, we perform a sensitivity check by replacing the binary dependent variable with a continuous one (i.e., the number of types of housing difficulty). The results by using the continuous variable of housing difficulties, as shown in the last column of Table 5, show that the coefficient of housing difficulties is negative and statistically significant at the 1% level. The results confirm that housing difficulties lead to lower life satisfaction, holding everything else constant.

## Conclusion and discussion

### A brief summary

This study shows strong evidence that households who experience housing difficulties are less satisfied with their lives than those who do not after controlling for a wide range of household demographic and socioeconomic characteristics and county and year fixed effects. Our estimated results are robust to unobservable household characteristics, model misspecification and selection bias. This result is consistent with the conclusions of few previous studies that show positive effects of housing conditions on wellbeing (Cattaneo et al., 2009; Zhang et al., 2018; Hu et al., 2020). Housing conditions and housing difficulties have similarities but they are not identical. This study complements the literature on the association between housing conditions and wellbeing because housing conditions are important criteria in determining whether there exists a housing difficulty.

We also provide explanations for the negative effects of housing difficulties on life satisfaction through which housing difficulties are detrimental to physical and psychological health. Indeed, our results show that households with housing difficulties are more likely to have poor physical health (i.e., self-reported physical health, experience of chronic disease and experience of bad memory) and psychological health (i.e., frequency of feeling depressed, frequency of feeling that everything is an effort and frequency of feeling hopeless). These findings are in line with life circumstance theory of life satisfaction, which proposes that life satisfaction can be explicitly considered as an overall judgement of life (Kusier and Folker, 2021). We need to indicate that life satisfaction remains negatively associated with housing difficulties even after controlling for health status in our empirical results. This finding implies the existence of other channels that housing difficulties affect life satisfaction, which is also expected because housing is associated with considerably various social and economic outcomes (Aaronson, 2000; Glaeser and Sacerdote, 2000; Dietz and Haurin, 2003; Engelhardt et al., 2010; Coulson and Li, 2013).

### Theoretical contributions and practical implications

This paper contributes to the literature in the following areas. First, we clarify housing difficulties as a new antecedent of individuals’ subjective wellbeing and thus enrich our understanding of the determinants of subjective wellbeing. Second, we add to the existing literature examining the outcomes associated with housing difficulties. Third, we identify possible mechanisms underlying the relationship between housing difficulties and household wellbeing and thus sheds light on the importance of housing problems.

The results in this study carry broad policy implications. Understanding the determinants of subjective wellbeing is important for the public and researchers, especially for public policy makers who are responsible for improving the wellbeing of the population. The literature documents a vast range of social, economic and political benefits brought by homeownership (Glaeser and Sacerdote, 2000; Dietz and Haurin, 2003; Engelhardt et al., 2010). The benefits associated with homeownership are often regarded as the justification for the tax treatment of housing or any subsidisation of homeownership (Coulson and Li, 2013). Overall, the consensus amongst social scientists is that homeownership is positively associated with one’s subjective wellbeing.

This study pays close attention to the effect of housing difficulties on the wellbeing of residents, a dimension that has not yet received sufficient attention in the research on the relationship between housing and wellbeing. Our results show that housing difficulties are detrimental to individuals’ health and life satisfaction, providing a justification for government involvement in home improvement, rather than merely in promoting homeownership.

### Limitations and future directions

This study is subject to three limitations, a few of which imply important future research directions. First, we only provide one potential mechanism through which housing difficulties negatively affect physical and psychological health. Our results also suggest that there exist other mechanisms underlying how housing difficulties may affect life satisfaction. A productive area for further research is to use additional data or qualitative methods to explore more explanations of the link between housing difficulties and wellbeing. Second, we do not consider whether the negative effects of housing difficulties apply to all individuals or only limited to specific groups. Empirical studies show that the effects of housing-related characteristics on life satisfaction vary amongst individuals with different age and income (Zhang et al., 2018). Third, we do not investigate the potential moderating effects of household and market factors on the relationship between housing difficulties and life satisfaction. For example, intra-family relationship significantly affects the economic pressure and psychological distress of family members (Chzhen et al., 2021). The association between housing difficulties and life satisfaction can be shaped by the intra-family relationship. Finally, the focus of our investigation is China, and prospective comparative studies across countries are warranted to validate our findings.

## Data availability statement

The raw data supporting the conclusions of this article will be made available by the authors, without undue reservation.

## Author contributions

All authors listed have made a substantial, direct, and intellectual contribution to the work and approved it for publication.

## Funding

This work was supported by the National Natural Science Foundation of China (Nos. 72104088, 72274176, 72074097, and 72174181), and the Key Project of Natural Science Foundation of Zhejiang Province (LZ20G030002).

## Conflict of interest

The authors declare that the research was conducted in the absence of any commercial or financial relationships that could be construed as a potential conflict of interest.

## Publisher’s note

All claims expressed in this article are solely those of the authors and do not necessarily represent those of their affiliated organizations, or those of the publisher, the editors and the reviewers. Any product that may be evaluated in this article, or claim that may be made by its manufacturer, is not guaranteed or endorsed by the publisher.

## Footnotes

^2^The CFPS is funded by the Chinese government through Peking University. The CFPS promises to provide to the academic community the most comprehensive and highest-quality survey data on contemporary China. The sample for the 2010 CFPS baseline survey through a multi-stage probability is drawn with implicit stratification. It is designed to be multi-stage so as both to reduce the operational cost of the survey and to allow for studies of social contexts. Each subsample in the CFPS study is drawn through three stages: county (or equivalent), then village (or equivalent), then household.

^3^Every household in China is required to have a registered residency with a local government authority, either urban hukou or rural hukou (Tang and Coulson, 2017). Many resources and benefits, including access to health care, free public education, housing and better access to jobs, are restricted to local residents with urban hukou (Au and Henderson, 2006; Glaeser et al., 2017; Gu et al., 2020,^2022^). Similar to registered residency, membership in the Chinese Communist Party has special rights and interests, including high-paying and prestigious jobs (Dickson and Rublee, 2000). Therefore, political status and hukou status are considered to be related to life satisfaction because of their connection to many benefits and resources.

^4^We compute variance inflation factors (VIF) after running Eq. (1) with the linear regression model. The highest VIF of the variables is 1.99, which is far below the generally acceptable level of 10.

^5^In order to understand the real effect of physical and psychological health, we use two steps as follows: First, we use principal component analysis for three physical (psychological) health variables to compress them into one variable that reflects the overall physical (psychological) health status. Second, we conduct the Sobel (1982) test on the significance of the mediation effect. Results of mediational models are reported in Appendix Tables 1, 2. Introducing the mediator somewhat reduces the value of the total effect, but it is still different from zero. It suggests that we only observe a partial transmission mediation effect. Moreover, both mediators passed the Sobel (1982), further supporting the premise that the mediator effect does exist. Based on our calculation, the proportion of mediator effects of physical (psychological) health in total effects is 5.7% (8.0%).
