# Supplementary material for: Housing difficulties, health status and life satisfaction
Source: Front Psychol. 2022 Dec 21;13:1024875. doi: 10.3389/fpsyg.2022.1024875 (PMC9811203; doi:10.3389/fpsyg.2022.1024875)
Supplement: Supplementary file 1 [file Data_Sheet_1.docx]

**Appendix**

**Table 1 ﻿Test the mediating effect of** **physical health**

|  | **Without mediator** | **With mediator** | |
| --- | --- | --- | --- |
|  | **(1): Life satisfaction** | **(2): Physical health** | **(2): Life satisfaction** |
| Housing difficulties | -0.162*** | 0.067*** | -0.153*** |
|  | (0.014) | (0.009) | (0.014) |
| Physical health |  |  | -0.137*** |
|  |  |  | (0.008) |
| Total effect | -0.162*** | | |
|  | (0.014) | | |
| Mediator effect | -0.009*** | | |
|  | (0.001) | | |
| Proportion ofmediator effect in total effect | 5.7% | | |
| Sobel (1982) | -0.009*** | | |
|  | (0.001) | | |
| **Other variables controlled** |  |  |  |
| Demographic characteristics | Yes | Yes | Yes |
| Socioeconomic status | Yes | Yes | Yes |
| Year dummies | Yes | Yes | Yes |
| County dummies | Yes | Yes | Yes |
| Pseudo R^2^ | 0.1423 | 0.1115 | 0.1115 |
| Observations | 39,987 | 39,987 | 39,987 |

Note: * significant at 10% level, ** significant at 5% level, *** significant at 1% level. Households demographic characteristics include gender, age, hukou status, political status and marital status. Household socioeconomic status includes education, insurance, household income, household deposit, finance assets value, housing assets value and household debts.

**Table 2 ﻿Test the mediating effect of psychological health**

|  | **Without mediator** | **With mediator** | |
| --- | --- | --- | --- |
|  | **(1): Life satisfaction** | **(2): Psychological health** | **(2): Life satisfaction** |
| Housing difficulties | -0.175*** | 0.081*** | -0.161*** |
|  | (0.015) | (0.012) | (0.015) |
| Psychological health |  |  | -0.172*** |
|  |  |  | (0.006) |
| Total effect | -0.175*** | | |
|  | (0.015) | | |
| Mediator effect | -0.014*** | | |
|  | (0.002) | | |
| Proportion ofmediator effect in total effect | 8.0% | | |
| Sobel (1982) | -0.014*** | | |
|  | (0.002) | | |
| **Other variables controlled** |  |  |  |
| Demographic characteristics | Yes | Yes | Yes |
| Socioeconomic status | Yes | Yes | Yes |
| Year dummies | Yes | Yes | Yes |
| County dummies | Yes | Yes | Yes |
| Pseudo R^2^ | 0.1423 | 0.1115 | 0.1115 |
| Observations | 39,987 | 39,987 | 39,987 |

Note: * significant at 10% level, ** significant at 5% level, *** significant at 1% level. Households demographic characteristics include gender, age, hukou status, political status and marital status. Household socioeconomic status includes education, insurance, household income, household deposit, finance assets value, housing assets value and household debts.
